# Comparative Utility of the Bromide and Iodide of Potassium, in the Treatment of Secondary and Tertiary Forms of Syphilis

**Published:** 1847-09

**Authors:** John Egan


*Comparative Utility of the Bromide and Iodide of Potassium, in the Treatment of Secondary and Tertiary forms of Syphilis.* By John Egan, M.D.—The high price which iodine has attained, has induced many practitioners to seek a less costly substitute, and bromine has been recommended for that purpose, and said to possess analogous properties. Dr. Egan, Surgeon to the Westmoreland Lock Hospital, has experimented pretty largely with this latter article, and in a paper read before the Surgical Society of Ireland, 1st May last, has given the results of his investigations, which by no means confirms the promises held out in its favor.

To form, says Dr. E., anything like a correct estimate of its effects, I selected for trial patients similarly affected with those with whom I had been accustomed to treat with the iodide of potassium, and have drawn up a statistical table of results, which has enabled me to institute a comparison between the two modes of treatment. The varieties of disease which I have arranged into four classes were as follows:—

1st. The eruptive form, comprising the papular, rupial, and scaly varieties.

2d. Affections of the throat, comprising increased vascularity, ulceration at back of pharynx, and excavated ulcer of the tonsil.

3d. Osteocopic pains.

4th. Ulcers of legs.

In the first or eruptive class there were eighteen cases of the papular variety, of which a cure was accomplished in fourteen after a protracted period. In the remaining four it appeared to exert no effect, and the iodide was eventually substituted.

In the two cases of rupia, one patient died, worn out by frequent epileptic attacks, in whom the bromide had been used with little benefit for six weeks. In the other, no perceptible alteration was manifested in the symptoms which presented.

In the scaly variety it failed in one; the other recovered after a lengthened period, desquamation appearing to be the result more of time than the operation of medicine.

In the second class, consisting of affections of the throat, its use was attended with success in two cases of increased vascularity in which it was employed: on one case of excavated ulcer of the tonsil (the only one from the rarity of this form of disease in which I had an opportunity of testing its effects), it produced no beneficial result; and out of six cases of ulcertion of the pharynx, its protracted use was only productive of advantage in three.

In eighteen cases of syphilitic pains, success followed in fourteen instances; in the remaining four the iodide was resorted to, to effect a cure. During its administration in this form of the disease, I was frequently obliged to have recourse to anodynes, in order to render the patient insensible to pain, over which for a considerable period it appeared to exert no salutary influence.

In the fourth class, comprising ulcers of the lower extremities, which might be more properly termed syphilitic cachexia, it produced a beneficial effect in two, but failed in three. The minimum dose employed in these several instances was five grains, the maximum ten, three times a day, beyond which I found it impossible to push it; the vehicle selected for its exhibition was water, with the addition of a little simple syrup.

In taking a retrospective view, extending over a period of four years, of the cases of secondary and tertiary syphilis treated with the iodide of potassium, and those just detailed in which the bromide was substituted, I think the former line of treatment must strongly recommend itself to every impartial mind, for the following reasons:—

First. The iodide exerts in the majority of instances an instantaneous, decided, and always a beneficial action, contrasted with the bromide, whose effects are slow, unsatisfactory, and frequently unsuccessful.

Secondly. The iodide seems to act favorably, not only upon the disease for which it is prescribed, but also upon the constitution in general, by increasing the appetite, improving the powers of digestion, thereby enabling the patient to gain flesh while under its influence, whilst the bromide not unfrequently produces nausea, impairs the appetite, and deranges the digestive organs.

And lastly, every form of secondary and tertiary syphilis (with the exception of iritis) is amenable to the action of the iodide, whilst that of the bromide is extremely circumscribed. A very general impression prevails among the profession that in order to obtain favorable results from the exhibition of the iodide of potassium, it is requisite to administer it in large doses. From the experience which I have had in its employment in the Lock Hospital, I should say that far more desirable consequences are likely to ensue from moderate than excessive doses; it has seldom occurred that every wished-for indication was not fulfilled by five grain doses, and in no instance did it appear necessary to increase it further than ten grains thrice a day.

The vehicle most commonly selected for the exhibition of iodide of potassium, which, by the majority of writers is considered materially to assist its therapeutic qualities, is some preparation of sarsaparilla, usually the compound decoction. From repeated experiments, I feel convinced that the beneficial effects of the iodide are in no way assisted by these preparations, as to the utility of which, either directly or indirectly, reasonable doubts may be entertained.

Dr. Stapleton confirmed Dr. Egan's views, and stated that, in comparison with the iodide of potassium, he had found the bromide almost inert.

Dr. Geoghegan has used the bromide pretty extensively, and also was led to a nearly similar estimate of its inefficacy. In some rupial affections, however, he had found it decidedly advantageous; and in venereal rheumatism, also, it had been extremely serviceable; but in most other respects its action was probably inferior to that of the hydriodate.—*Dublin Med. Press.—Am. Jour. Med. Sci.*

